# Function and regulation of RGS family members in solid tumours: a comprehensive review

**DOI:** 10.1186/s12964-023-01334-7

**Published:** 2023-11-03

**Authors:** Chenglong Yang, Xiaoyuan Zhang, Xiaowen Yang, Fuming Lian, Zongrun Sun, Yongming Huang, Wenzhi Shen

**Affiliations:** 1 Key Laboratory of Precision Oncology in Universities of Shandong, Institute of Precision Medicine, Jining Medical University, Jining, 272067 China; 2 Department of General Surgery, Affiliated Hospital of Jining Medical University, Jining Medical University, Jining, 272067 China

**Keywords:** RGS family, Structure, Tumour, Function, Signalling pathways, Biomarker

## Abstract

G protein-coupled receptors (GPCRs) play a key role in regulating the homeostasis of the internal environment and are closely associated with tumour progression as major mediators of cellular signalling. As a diverse and multifunctional group of proteins, the G protein signalling regulator (RGS) family was proven to be involved in the cellular transduction of GPCRs. Growing evidence has revealed dysregulation of RGS proteins as a common phenomenon and highlighted the key roles of these proteins in human cancers. Furthermore, their differential expression may be a potential biomarker for tumour diagnosis, treatment and prognosis. Most importantly, there are few systematic reviews on the functional/mechanistic characteristics and clinical application of RGS family members at present. In this review, we focus on the G-protein signalling regulator (RGS) family, which includes more than 20 family members. We analysed the classification, basic structure, and major functions of the RGS family members. Moreover, we summarize the expression changes of each RGS family member in various human cancers and their important roles in regulating cancer cell proliferation, stem cell maintenance, tumorigenesis and cancer metastasis. On this basis, we outline the molecular signalling pathways in which some RGS family members are involved in tumour progression. Finally, their potential application in the precise diagnosis, prognosis and treatment of different types of cancers and the main possible problems for clinical application at present are discussed. Our review provides a comprehensive understanding of the role and potential mechanisms of RGS in regulating tumour progression.

Video Abstract

**Supplementary Information:**

The online version contains supplementary material available at 10.1186/s12964-023-01334-7.

## Background

Cancer is one of the leading causes of human death worldwide. According to GLOBOCAN data released in 2021 by the International Agency for Research on Cancer, a division of the World Health Organization, there were approximately 19.3 million new cancer cases and approximately 10 million deaths worldwide in 2020 [1]. Cumulative alterations in genome structure and function drive the development of cancer [2]. With the deepening understanding of tumours, their key characteristics have been generalized and are constantly being updated [3]. At the same time, new tumour-associated markers and their mechanisms of action are being discovered. Therefore, it is essential to summarize the typical mechanisms and newly discovered pathways of tumour-associated proteins mediating cancer progression so that more targeted interventions can be implemented to control cancer progression, further reducing cancer mortality and prolonging patient survival.

**Fig. 1:**
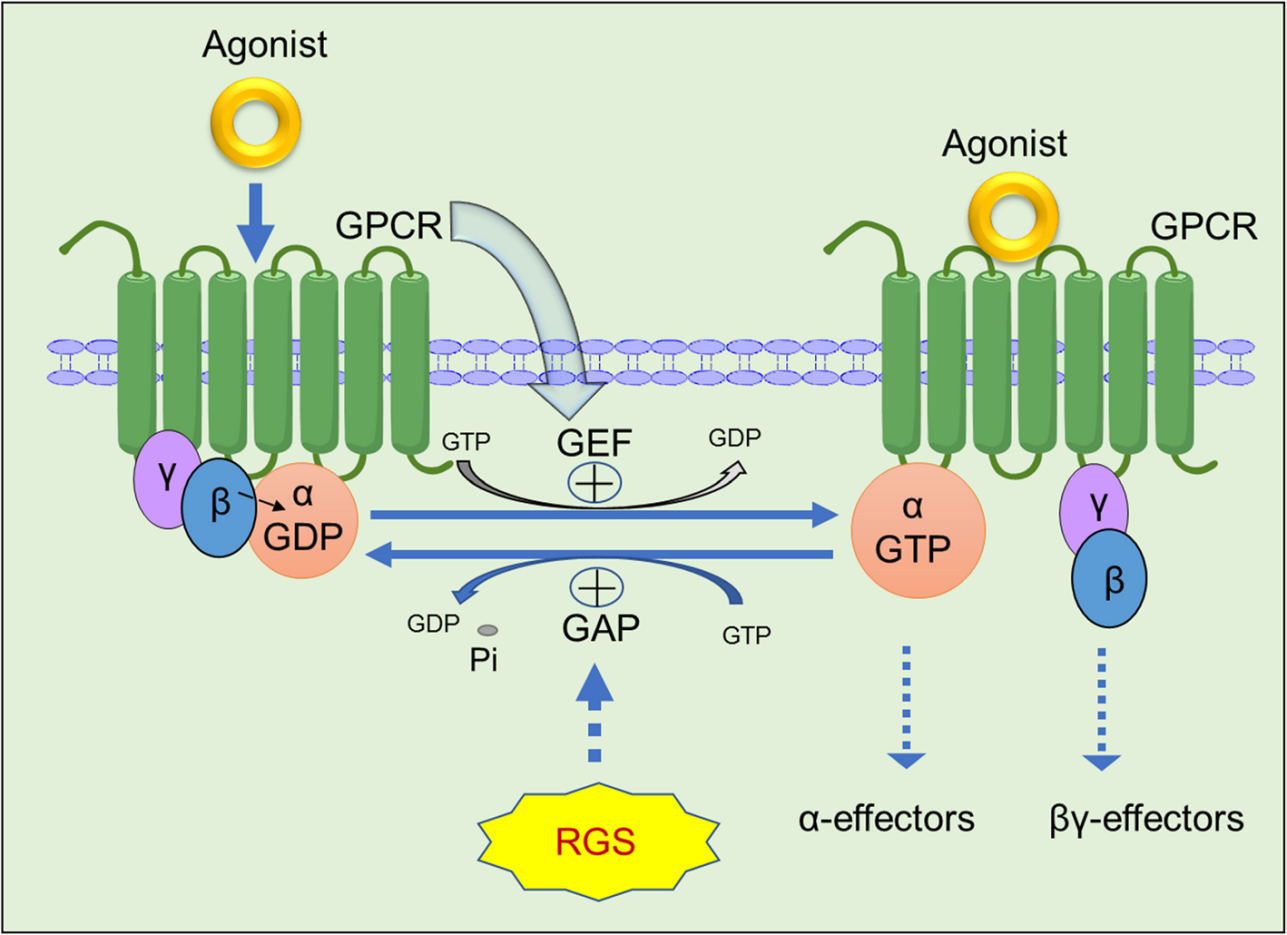
The canonical regulation pattern of GPCR signalling by RGS proteins. When G protein-coupled receptors (GPCRs) specifically recognize and bind to agonists, GPCRs cause conformational changes that promote the activation of Gα-GDP on the α subunit of the heterotrimer complex in exchange for free GTP, plus the release of Gβγ dimers that can all continue to conduct some downstream signals or effectors. RGS proteins are Gα-GTP hydrolase accelerator proteins (GAPs) that can terminate the signal transduction of GPCRs by promoting Gα-GTPase activity and GTP hydrolysis inactivation after interchanging with GDP and promoting the heterotrimer complex Gβγ to recombine with the receptor on the cell membrane

Previous studies have shown that G protein signalling regulator (RGS) proteins can participate as GTP hydrolases (GAPs) in the recycling process of Gα-GDP and Gα-GTP in heterotrimers (GPCRs). In the absence of agonists, the α-subunit of the seven-transmembrane G protein binds to GDP to form Gα-GDP, which binds to the Gβγ heterodimer to form the Gα-GDP/Gβγ closed-cycled heterotrimer. The heterotrimer complex further interacts with the G protein-coupled receptor (GPCR). In this process, the binding of GDP to Gα attenuates the spontaneous interchange activity of GDP with GTP. In contrast, the Gβγ heterodimer promotes the coupling of Gα to GPCRs. Upon binding with an agonist, GPCRs undergo a conformational change that facilitates the exchange of GDP for GTP on the Gα subunit of the heterotrimeric complex. Both GTP-bound Gα in the active form and the released Gβγ heterodimer can subsequently stimulate the corresponding downstream signalling. When GAP is present, it can promote the hydrolysis of small phosphate groups in Gα-GTP and interchange with GDP to form Gα-GDP again and then resume the change process into the Gα-GDP/Gβγ heterotrimer. The RGS family can act as a GAP instead of regulating the enzymatic reaction between GDP and GTP [4–6] (Fig. 1) [7].

In recent years, GRCRs have been implicated in the development of a wide range of diseases. RGS proteins, as key regulators of GPCR signalling, may also play an important role in modulating the pathophysiological progression of many types of diseases. The RGS superfamily contains a number of regulators that bind to Gα through the "RGS box" domain (also known as the RH domain), which contains a 120 amino acid signature [7]. In cancer progression, these RGS proteins act as gating switches that are critical for regulating tumour cell growth, proliferation, differentiation, and migration [5, 8, 9]. However, there are very limited systematic reviews on the functional/mechanistic characterization and clinical applications of RGS family members in tumours at present.

In this review, we provide an overview of recent reports on individual members of the RGS family, summarize the history and structure of RGS and its role in cancer, and further discuss the molecular mechanisms that govern RGS protein expression, providing insights into future novel targeted drug development and related cancer therapies.

### Classification, structural domains and regulation of the classical RGS protein family in cancer

**Fig. 2:**
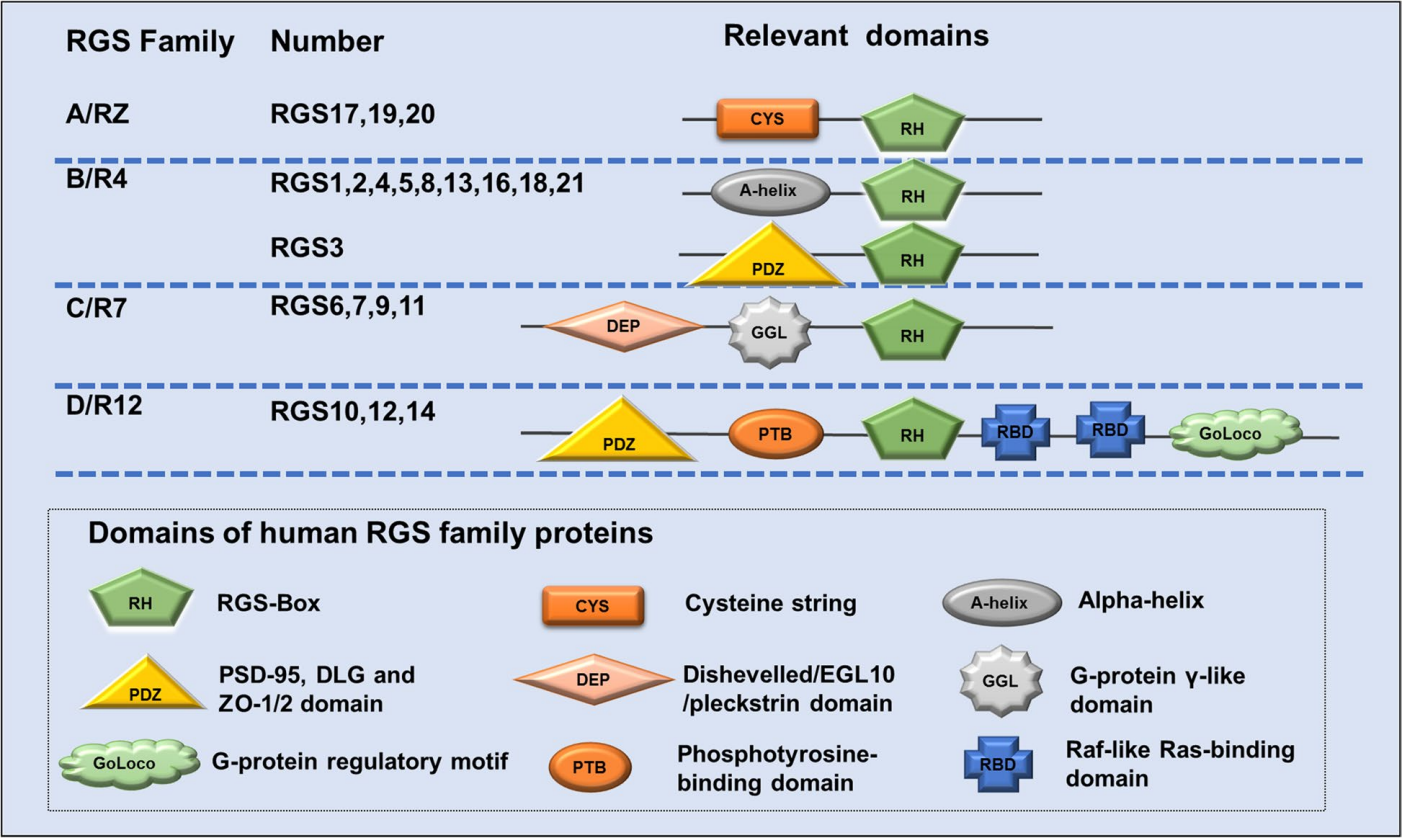
Classical RGS subfamily and related structural domains. Based on the RGS homology and structural domains, the classical RGS proteins were divided into four subfamilies. The four subfamilies are RGS A/RZ, B/R4, C/R7 and D/R12, and the members of each subfamily are also listed in the figure. Each subfamily contains a G protein-specific RGS domain (RH), the "RGS Box", which acts on the Gα subunit and exhibits GAP activity, in addition to the cysteine string (CYS) in the RGS A/RZ subfamily. R4 subfamily RGS3 also has the domains of PSD-95, Dlg, and ZO-1/2 (PDZ); the R7 subfamily also contains the Dishevelled/EGL10/pleckstrin domain (DEP) and G-protein γ-like domain (GGL); and the R12 subfamily contains the phosphotyrosine-binding domain (PTB), Raf-like Ras-binding domain (RBD) and G-protein regulatory motif (GoLoco) in addition to the PDZ structural domain

Mammalian G protein signalling proteins (RGS) contain more than 20 family members. Based on sequence homology and different protein structural domains, the traditional RGS family can be divided into four subfamilies, RGS A/RZ to RGS D/R12. Each subfamily contains multiple members, and each RGS protein contains one or more structural domains of approximately 120 amino acids (RH), called RGS boxes, that are responsible for regulating the activity of GTPase-activating protein (GAP). In turn, GAP is a key regulatory point in the GPCR cycle that promotes G protein inactivation. Thus, the RGS protein greatly enhances the action of GAP, further increasing GTPase activity by a thousand-fold (Fig. 2) [10].

### RGS A/RZ subfamily

**Fig. 3:**
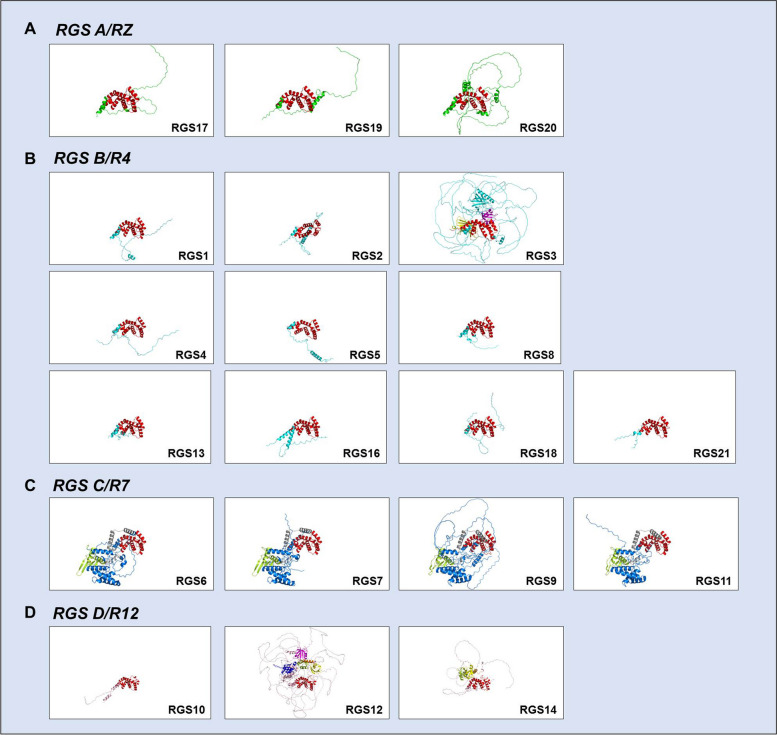
Basic structure and different structural domains of RGS proteins. **A** Basic structural domains of the RGS A/RZ subfamily, which contains RGS 17, RGS 19 and RGS 20. Red: RGS-Box (RGS domain). **B** Basic structural domains of the RGS B/R4 subfamily, which contains RGS 1–5, RGS 8, RGS13, RGS16, RGS18 and RGS 21. Red: RGS-Box (RGS domain). For RGS3, yellow: C2 domain, red: RGS-Box, purple: PDZ domain. **C** Basic structural domains of the RGS C/R7 subfamily, which contains RGS6, RGS7, RGS9 and RGS11. Green: DEP domain, grey: G protein domain, red: RGS-Box. **D** Basic structural domains of the RGS D/R12 subfamily, which contains RGS10, RGS12 and RGS14. Purple: PDZ domain, blue: PID domain, red: RGS-Box, green: RBD1 domain, yellow: RBD2 domain, orange: Goloco domain

**Fig. 4:**
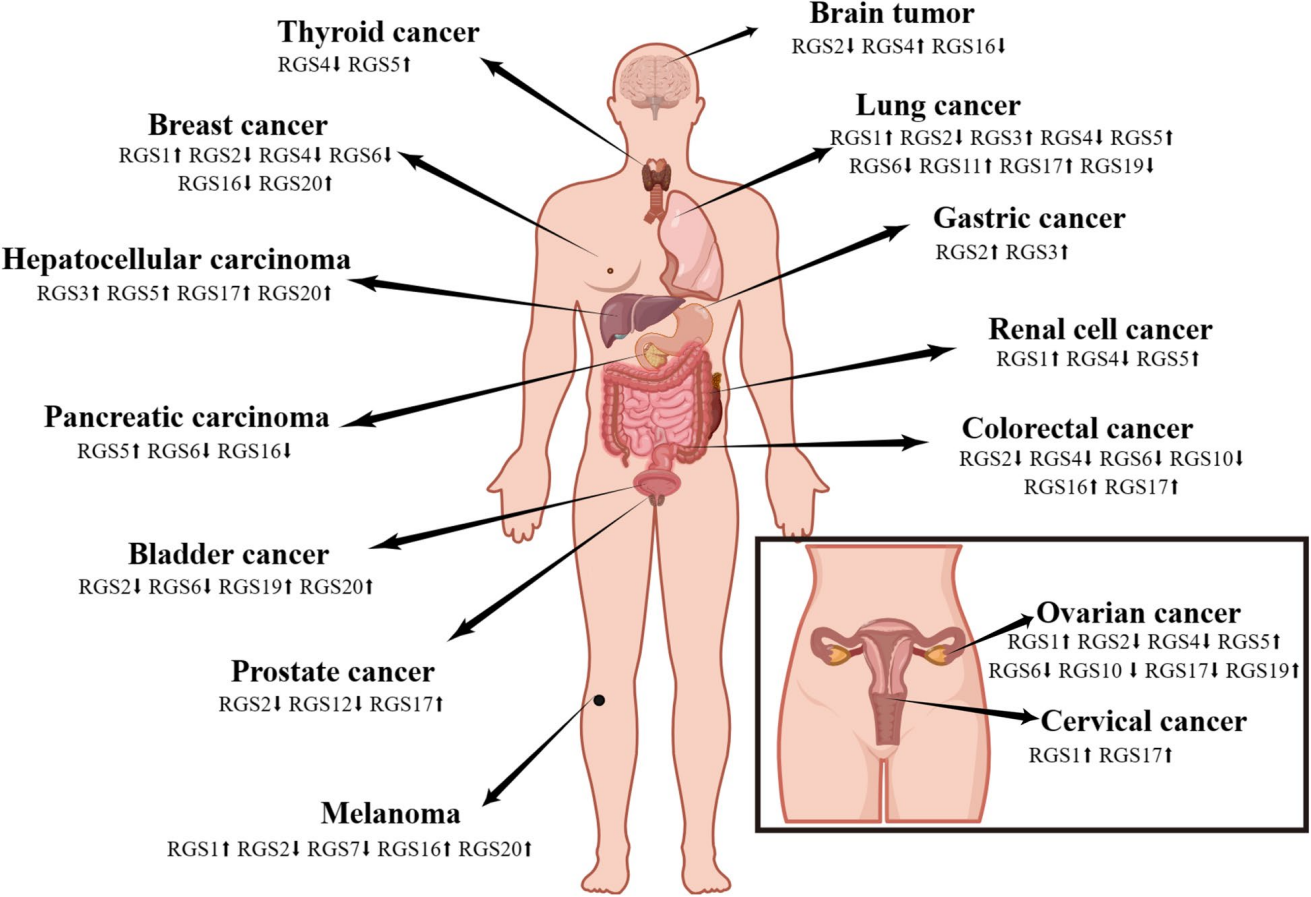
Overview of RGS family protein expression in various human tumour types. Arrows indicate the regulation in the respective tumour cells. The main concern is the regulation of RGS in the tumours described in this review (brain, lung, gastric, renal cell, colorectal, ovarian, cervical, thyroid, breast, hepatocellular, pancreatic, bladder, prostate, and melanoma). This figure was created with permission and drawn by Figdraw

**Fig. 5:**
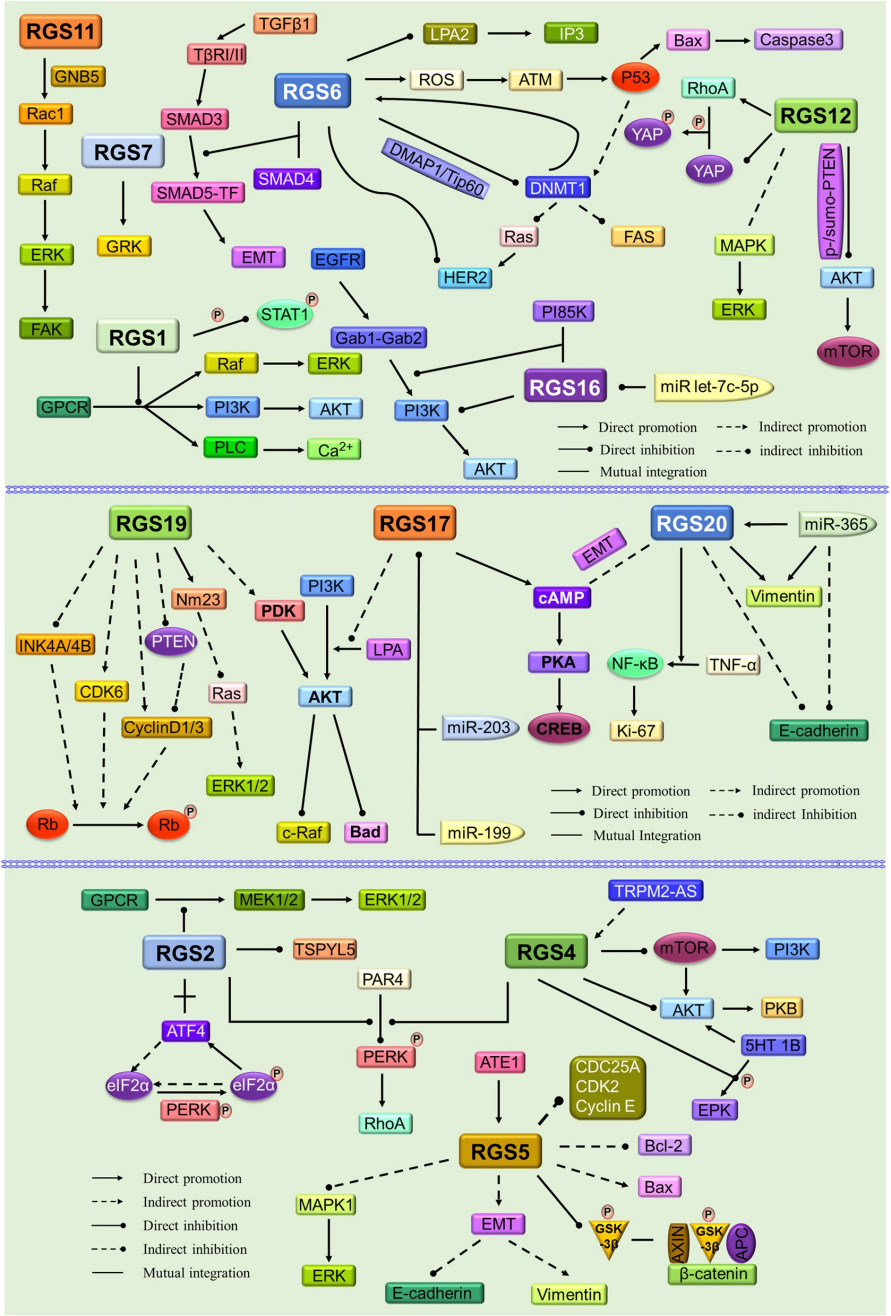
Schematic diagram of the relevant signalling pathways in which RGS family members function

The RGS A/RZ subfamily consists mainly of three members, RGS17, RGS19, and RGS20, all of which are small and simple proteins with the major associated structural domain cysteine string (CYS) located near the N-terminus, which is used mainly to regulate the membrane localization of RGS proteins (Figs. 2 and 3A). It is used mainly to regulate membrane localization and interacts with other components as a binding site and is small in molecular weight but conserved and stable [11]. Garnier et al. found that RGS17, also known as RGSZ2, is expressed almost exclusively in the brain, with little expression in other tissues of the body [12]. However, in the pathological state of cancer, it is significantly expressed in a variety of tissues. In particular, through methods such as high-throughput screening, many studies have identified RGS17 as a new target in lung and prostate cancers, and the specific mechanism may be related to the induction of tumour cell proliferation by RGS17 through regulation of the cAMP-PKA-CREB pathway [13–16]. It was also reported that RGS17 was aberrantly highly expressed in colorectal, hepatocellular and cervical cancer tissues and their cell lines [17–19], but interestingly, it is markedly reduced in ovarian cancer and appears to function as a tumour suppressor gene, which may be due to the inhibition of the LPA-mediated AKT activation pathway by the expression of RGS17 [20] (Figs. 4 and 5, Table 1).

**Table 1:** RGS family members as potential biomarkers and their role in different tumour types

RGS family	RGS member	Tumour	Expression (Compare to normal)	Effects
RGS A/RZ	RGS17	Non-small cell lung cancer	Up	Cell proliferation, regulation of the cAMP-PKA-CREB pathway [15, 16] and miRNA-199, miR-203 inter-regulation [21, 22]
		Prostate cancer	Up	Inhibition of AMP kinase, promoting development [13, 15, 16] and miR-203 interactions affecting cell proliferation invasion and migration ability [23]
		Ovarian Cancer	Down	Regulation of LPA-mediated AKT activation process [20]
		Cervical cancer	Up	Positive correlation with Lincoo483, promotes proliferation and invasion, inhibits apoptosis [19]
		Hepatocellular carcinoma	Up	Regulation of cancer cell proliferation, migration and invasion [18]
		Colorectal cancer	Up	Promotes growth and migration [17]
	RGS19	Ovarian cancer	Up	Attenuates cell cycle control and enhances AKT signalling [24]
		Bladder cancer	Up	Significant effect on survival rate [25]
		Non-small cell lung cancer	Down	Inhibition of Ras activation-induced tumour formation [26]
	RGS20	Breast cancer	Up	Downregulation of E-calmodulin expression, promoting metastasis [27]. Promotes lymph node metastasis, cancer progression, and is associated with poor prognosis [28]
		Ovarian cancer	ND	Involvement in MAPK, AKT signalling pathways [29]
		Bladder cancer	Up	Activation of NF-κB signalling is associated with cell proliferation migration, overall survival [30]
		Hepatocellular carcinoma	Up	Associated with lincRNAs with oncogenic potential role [29]
		Melanoma	Up	Downregulation of E-calmodulin expression promotes metastasis [27]
		Oral cancer	Up	Reversal of miR-365, Enhances cell viability and motility [31]
RGS B/R4	RGS1	Breast cancer	Up	Affects inwards flow of calcium, and activation of ERK and AKT kinases, affects cAMP levels, regulates inwards flow of calcium, and immune escape [32, 33]
		Ovarian cancer	Up	Associated with immune infiltration [34]
		Cervical cancer	Up	Affects T-cell activation and significantly correlates with immune infiltration and ICI target expression [35]
		Non-small cell lung cancer	Up	Affects inwards flow of calcium, activation of ERK and AKT kinases, immune escape [33]
		Melanoma	Up	Value-added migration: with tumour thickness, mitotic rate, presence of damaged vessels; anterior lymph node metastasis [36, 37]
		B-cell lymphoma	Up	Impact on overall survival [38]
	RGS2	Breast cancer	Down	Mediates the MCPIP1 pathway to inhibit growth [39]. Negatively correlated with miR-183-5p [40] Mediated Slug regulates epithelial-mesenchymal transition [41]
		Bladder cancer	Down	Inhibition by UHRF1 is associated with cell proliferation [42] Regulation by ZHX3 affects the migration and invasive ability of tumour cells [43]
		Ovarian cancer	Down	Epigenetic changes related to histone modifications and DNA methylation [44]. Regulation of lipopolysaccharide-mediated downstream signalling [45]
		Prostate cancer	Down	Growth inhibitory factor, ERK 1/2 is involved; affects androgen-independent tumour cell growth [46, 47]
		Non-small cell lung cancer	Down	Degrading transcription factors, a biomarker of proliferative retardation and poor prognosis [48]
		Stomach cancer	Up	Significant association with CD8 + T-cell infiltration [49]
		Colorectal cancer	Down	Participates in ERK phosphorylation, regulates Rho activity, and affects cell proliferation [50, 51]; has a role in cancer metastasis [52]
		Melanoma	Down	Partial effect antagonist, affecting cell proliferation; [53] inhibits MAPK and AKT pathways [54]
		Oral cancer	Down	Associated with proliferation of lymphovascular invading cells [55]
		Glioblastoma	Down	Stress [56], cell proliferation, migration and invasion are affected by TRPM2-AS expression [57]
	RGS3	Non-small cell lung cancer	Up	Negatively correlated with miR-25 and influences apoptotic cell death [58]
		Stomach cancer	Up	Negatively regulates microRNA-133a and affects cell proliferation [59]
		Hepatocellular carcinoma	Up	Promotes value addition and inhibits apoptosis [60]
	RGS4	Breast cancer	Down	Affects pseudopod formation, affects G protein-coupled receptor signalling, and is associated with migratory invasion [61–63]
		Ovarian cancer	Down	Negative regulation that attenuates LPA-stimulated cell signalling [64]
		Thyroid cancer	Down	Interacts with miR-3663-3p and is involved in cell proliferation, apoptosis [65]
		Colorectal cancer	Down	Participates in ERK phosphorylation, regulates Rho activity, and affects cell proliferation [50]
		Renal cell carcinoma	Down	Associated with P16 and poor prognosis [66]
		Non-small cell lung cancer	Down	Proliferation is positively correlated, but migration is not; [67] negative correlation with lymph node metastasis and TNM staging [62]
		Glioblastoma	Up	Affects the invasion and migration ability of cancer cells and induces apoptosis [68] Regulates the mTOR signalling pathway [69]
		Neuroblastoma	Down	Inhibits 5-HT (1B) receptor coupling; inhibits Akt pathway [70] regulates δ-opioid receptor signalling [71]
	RGS5	Ovarian cancer	Up	Hypoxia reduces the MAPK/ERK signalling pathway [72] and affects the angiogenic microenvironment [73]
		Thyroid tumours	Up	Physiological modulators of calcium-sensitive receptors [74]
		Parathyroid tumour	Up	Inhibition of signalling at calcium-sensitive receptors [75]
		Non-small cell lung cancer	Up	Associated with invasion and metastasis; [76] induces apoptosis and affects adhesion capacity [77]
		Hepatocellular carcinoma	Up	Induction of epithelial-mesenchymal transition is associated with hepatocyte injury and fibrosis [78] and is involved in the regulation of GSK-3β activity and Wnt/β-catenin signalling [79]
		Renal cell carcinoma	Up	Involved in GPCR-mediated signalling [80] and affects angiogenesis [81]
		Stomach cancer	ND	Positively correlated with tumour differentiation and negatively correlated with MVD [82, 83]
		Pancreatic cancer	Up	Pericyte markers that affect the normalization of the tumour vascular system [84]
	RGS8	ND	ND	ND
	RGS13	B-cell lymphoma	Up	A possible novel marker for MCL [85]
	RGS16	Breast cancer	Down	Attenuates phosphatidylinositol 3-kinase signalling, affects cell proliferation [86] and is negatively correlated with tumour cell aggressiveness [87]
		Colorectal cancer	Up	Prognostic markers [88]
		Melanoma	Up	Negatively correlated with T-cell stemness-related genes [89]
		Pancreatic cancer	Down	Interaction with FosB affects lymph node metastasis and overall survival [90]
		Chondrosarcoma	ND	Negatively correlated with miR-181a and growth, angiogenesis and metastasis [91]
		Glioblastoma	Down	Activation of the PI3K-AKT pathway affects survival, and epithelial-mesenchymal transition is significantly associated with poor prognosis [92]
	RGS18	ND	ND	ND
	RGS21	ND	ND	ND
RGS C/R7	RGS6	Breast cancer	Down	Activates apoptosis, involved in Bax/Bcl-2, P53 pathway [93, 94], promotes cell apoptosis and inhibits cell growth [95]
		Bladder cancer	Down	Tumour suppressors that promote P53 activation and DNMT1 downregulation [96–98]
		Ovarian cancer	Down	Negative regulation that attenuates LPA-stimulated cell signalling [64]
		Non-small cell lung cancer	Down	Interacts with SMAD4 and inhibits epithelial-mesenchymal transition [97]
		Colorectal cancer	Down	Associated with CEA levels, TNM staging, and lymphatic metastasis [99]
		Pancreatic cancer	Down	Associated with tumour differentiation, pT classification, and survival [100]
	RGS7	Melanoma	Down	Inhibitory factor, associated with tumour cell anchor growth, migration [101]
	RGS9	ND	ND	ND
	RGS11	Non-small cell lung cancer	Up	Biomarkers, which play an important role in cancer-related metastasis [102] are associated with advanced and aggressive cancer [103]
RGS D/R12	RGS10	Colorectal cancer	Down	There is a negative correlation with DNA methylation [104]
		Ovarian cancer	Down	Antagonizes mTOR signalling, cancer cell viability [105, 106]. Related to histone deacetylation and DNA methylation [107, 108]
		Neuroblastoma	ND	Involved in the regulation of AKT signalling pathway in relation to cellular self-viability [105]
	RGS12	Prostate cancer	Down	Negative regulation of AKT and MNX1 pathways [109]
		Oral cancer	Down	Interferes with PTEN phosphorylation and ubiquitination-like modifications that affect cell proliferation and migration [110]
		Osteosarcoma	Down	Inhibitory factor, inhibits tumour metastasis [111]
	RGS14	ND	ND	ND

*ND* Not determined

RGS19 is less known than RGS17, and this may be related mainly to its low expression level in the normal homeostasis of the organism. In a previous study, Michael E. et al. found that RGS19 can regulate Wnt-β-catenin signalling by inactivating Gα0, which is involved in numerous life processes in organisms [112], among which Wnt-β-catenin signalling can promote heart formation and cardiomyocyte differentiation in mice. However, in RGS19-overexpressing P19 teratoma cells, RGS19 inhibited cardiomyocyte differentiation by blocking Wnt signalling. By further studying RGS19-overexpressing transgenic mice (RGS19TG), Young Rae Ji et al. demonstrated that RGS19 affects cardiac development and negatively regulates cardiac function [113]. During the development of human solid tumours, RGS19 appears to promote the proliferation of tumour cells in situ while inhibiting the migratory development of tumour cells. According to The Cancer Genetic Atlas (TCGA) and DepMap databases, RGS19 has been reported to be overexpressed in numerous cancers, particularly in bladder and ovarian cancers, where RGS19 can achieve unrestricted cell proliferation by enhancing AKT signalling and cell cycle control of the immune system. RGS19 also regulates the cAMP/PKA/CREB pathway and transcriptionally upregulates the tumour metastasis suppressor Nm23, thereby attenuating the migration ability of tumour cells (Figs. 4 and 5, Table 1). Beyond this, there may be more complex mechanisms that have not been reported and that may require further exploration and study [24, 25, 114].

The RGS A/RZ subfamily, also known as RGSZ1 or Ret RGS in addition to RGS17 and RGS19, selectively binds and functions with Gαi2 and Gαz subunits. Its expression is similar to that of RGS19, with little or no expression in normal organisms. However, according to recent reports, RGS20 has been found to be significantly more highly expressed in various cancer tissues than in adjacent normal tissues, such as breast cancer, metastatic melanoma, hepatocellular carcinoma, and bladder cancer. Li Gang et al. found that RGS20 could activate NF-κB signalling through a series of biochemical assays, such as MTT, anchorage-independent growth assays, luciferase activity assays and related animal models. By overexpressing and knocking down RGS20 in different human cancer cell lines, Lei Yang et al. found that RGS20 could increase the expression of metastasis-related markers and downregulate the expression of adhesion proteins, which could indicate that RGS20 expression could promote cell proliferation and enhance the invasive migration ability of cancer cells [27, 29, 30] (Figs. 4 and 5, Table 1).

### RGS B/R4 subfamily

The RGS B/R4 subfamily is the most abundant member of these four subfamilies, including RGS1-5, RGS8, RGS13, RGS16, RGS18, and RGS21. All of these members are between 20–30 KD in size, except for RGS3, which has a larger molecular weight. The reason for the large molecular weight of RGS3 is that its structural domain is composed of PDZ [115] (Figs. 2 and 3B). Although the molecular weight of R4 family members is generally small, surprisingly, the major structural domain of these proteins, the "RGS Box", is able to recognize and bind different small subunit conformations of Gα to classify them [116], which is an important regulatory point for the participation of R4 members in the GPCR cycle. As the tissue distribution of each member of the RGS R4 subfamily and its relationship with physiology and disease have been reported in many studies previously [115, 117], we will mainly summarize its relationship with the development of each solid tumour here.

RGS1, an important member of the R4 RGS subfamily, has been shown to be associated with a variety of B-cell activation and B-cell chemokine regulatory induction signals [118], which are involved mainly in the immune response, interfering with the normal clearance function of lymphocytes, creating an immune escape, and providing a favourable microenvironment for the development of tumour cells [32]. However, the exact mechanism is not well understood. RGS1 is significantly upregulated in a variety of solid tumours, including renal cell tumours, melanoma, ovarian cancer, and cervical cancer, among others [119]. It was demonstrated that in melanoma, RGS1 can regulate Gαs-mediated phosphorylation of AKT and ERK to promote melanoma development; however, interestingly, this regulation is not involved in the hydrolysis process of GTP in GPCRs, and it has a non-GAP function [36, 37]. Moreover, Javier Rangel et al. also reported that upregulation of RGS1 expression was associated with increased tumour thickness and increased mitotic rate. Although the exact mechanism of action remains to be discovered, it is certain that RGS1 promotes tumour cell proliferation, migration and invasion and is associated with poor prognostic survival in diffuse large B-cell lymphoma and multiple myeloma [38, 120] (Figs. 4 and 5, Table 1).

In comparison to RGS1, RGS2 shows some differences. The expression of RGS2 is generally downregulated in most solid tumours, which is very different from the expression of RGS1. RGS2 specifically recognizes and prefers binding of Gαq subunits over other family members for GAP action [45]. Therefore, RGS2 is more characteristic of inhibiting cancer development than other members. The expression of RGS2 mRNA in breast cancer tissues is lower than that in the normal group, the expression of RGS2 in cancerous breast cells is also lower than that in normal breast cells, and its overexpression can inhibit the growth of breast cancer cells, although this mechanism needs to be further explored [39]. RGS2 protein expression is reduced in human prostate cancer specimens compared to adjacent normal or hyperplastic tissues, and RGS2 can regulate ERK1/2-mediated androgen-independent androgen receptor (AR) activation. Based on this, X Cao et al. suggested that RGS2 could act as a growth inhibitor for androgen-independent prostate cancer cells [46, 121]. It has also been reported that RGS2 expression could promote the migration and invasive ability of bladder uroepithelial carcinoma, and that inhibition of RGS2 expression in bladder uroepithelial carcinoma provides a promising target for the treatment of cancer [43]. However, RGS2 indicates the opposite in other tumour cancer types, and Yang S. et al. demonstrated elevated expression levels of RGS2 in gastric cancer cells by protein blotting and immunofluorescence staining. Pancancer analysis also showed that RGS2 was significantly associated with TMB, TID and CD8 + T-cell infiltration in other cancer types [49] (Figs. 4 and 5, Table 1).

RGS3 differs from other members of the R4 family in that, in addition to the "RGS Box" box, it has a PDZ structural domain at its C-terminus, which allows RGS3 to bind to GSK3β and inhibit its activity, enhancing the Wnt β-Catenin signalling pathway and thus promoting epithelial-to-mesenchymal transition (EMT) [122]. EMT is closely related to the aggressiveness and stem cell properties of cancer cells [123]. Briefly, RGS3 enhances the invasive and stem cell properties of cancer cells and interacts with noncoding small RNAs, which are important for the development of tumour cells. For example, miR-25 is negatively correlated with RGS3 expression, and its interaction is involved in the regulation of cancer cell stemness in non-small cell lung cancer [58]. MiR-145-5p and RGS3 are positively promoted in hepatocellular carcinoma and are important in promoting cell proliferation and inhibiting apoptosis [60]. In addition, microRNA-133a is also negatively correlated with RGS3 levels in gastric cancer, with significantly higher expression of RGS3 in gastric cancer cells and tissues than in corresponding normal tissues and cells [59] (Fig. 4, Table 1).

RGS4 is also a negative regulator of GPCRs, which can block relevant signalling by accelerating the hydrolysis of active Gα-GTP. RGS4 can form complex signalling molecule transduction complexes with different receptors, effectors, scaffolding proteins and other signalling molecules, affecting the localization, activity and stability of signals in cells and playing an important regulatory role in tumour tissues or cells [61]. According to Cheng Chuanle et al., the expression of RGS4 was higher in normal lung tissues than in non-small cell lung cancer specimens, and correlation analysis showed that the expression level of RGS4 was negatively correlated with lymph node metastasis and TNM stage, leading to RGS4 being considered a novel tumour suppressor. In a nude mouse metastasis model, overexpression of RGS4 was shown to inhibit the metastatic process of tumours in vivo [62]. This protein is also significantly expressed in normal breast epithelial cells, and silencing of RGS4 in breast cancer cells enhances the invasive ability of cancer cells. Mu Xianmin et al. found that targeted drugs, while increasing the expression of RGS4, inhibit the formation, migration and invasion of plate-like pseudopods in breast cancer cells, the underlying mechanism of which has not been elucidated [61]. In addition, RGS4 exhibits features in other tumour types that do not share the abovementioned functions. For example, in osteosarcoma tumour tissues, RGS4 interacts with a noncoding RNA (miR-874-3p) and affects the value-added and migration of cancer cells, and RGS4 overexpression promotes the value-added and migration of human osteosarcoma cells [124] (Figs. 4 and 5, Table 1).

RGS5, a protein that promotes apoptosis and resists tumour cell proliferation, is also a member of the RGS R4 family, is involved in the negative regulation of the GPCR cycle and is a hallmark molecule of tumour-associated pericytes; however, in the tumour microenvironment, proapoptotic RGS5 can be restricted by other regulatory signals or can even be converted to antiapoptotic RGS5 to enhance pericyte survival, and high expression in several cancers is associated with poor tumour growth and prognosis [125, 126]. RGS5 is highly expressed in most hepatocellular carcinoma tissue samples and cell lines. Hu et al. showed that knockdown of RGS5 expression significantly inhibited the migration and invasive ability of hepatocellular carcinoma cells, while overexpression promoted the development of epithelial-mesenchymal transition in hepatocellular carcinoma cells [127]. There is evidence that RGS5 may be involved in the regulation of GSK-3β activity and Wnt/β-catenin signalling, affecting the development of hepatocellular carcinoma [79]. Moreover, Dan Wang et al. showed that RGS5 was abundantly expressed in epithelial ovarian cancer compared to normal ovarian tissue, especially in the cytoplasm and microvascular structures (Figs. 4 and 5, Table 1). However, the underlying mechanism is unclear and may be related to the involvement of RGS5 in the regulation of angiogenesis [72].

RGS16 is one of the major oncogenes of the R4 family and promotes the malignant development of various human tumours [87]. Ruoyu Huang et al. demonstrated that RGS16 expression was positively correlated with glioma grade and that overexpression of RGS16 was closely associated with cell proliferation, migration, epithelial-mesenchymal transition, and immune and inflammatory responses in gliomas [128]. In addition, the mRNA and protein levels of RGS16 were reported to be higher in colorectal cancer tissues than in the corresponding normal tissues; therefore, RGS16 may be considered a predictive marker for cancers such as colorectal and pancreatic cancers [88, 90]. There is also evidence that inhibition of RGS16 can directly or indirectly enhance the migration and invasive ability of breast cancer cells, but the underlying mechanisms remain unclear [87] (Figs. 4 and 5, Table 1).

### RGS C/R7 subfamily

The structural domains of the RGS C/R7 family contain not only the "RGS Box" (RH) structure but also the Dishevelled/EGL10/Pleckstrin domain (DEP) and the G-protein γ-like domain (GGL). The DEP domain binds syntaxin-like proteins such as R7 binding protein (R7BP) to mediate intracellular localization, and the GGL domain can bind the G_B5_ subunit [5] (Figs. 2 and 3C). The R7 subfamily contains four major members, RGS6, RGS7, RGS9 and RGS11. The RGS6 structural domain is responsible for the GAP activity of RGS6 and other RGS proteins and allows it to negatively regulate the Gαi/o protein subunit, which is specifically involved in the development and progression of many cancer types [95]. In colorectal cancer and ovarian cancer, both RGS6 mRNA and protein expression are decreased, which is closely correlated with tumour size, CEA level, and TNM stage and is more prone to distant metastasis in lymph nodes [99]. RGS6 is associated with apoptosis, mediates apoptosis and cardiomyopathy induced by chemotherapeutic agents (adriamycin), etc., and is associated with poor prognosis in patients with pancreatic cancer [129, 130]. Interestingly, RGS6 has also been shown to regulate G protein-independent signalling. For example, in breast cancer, RGS6 promotes the degradation of the DNA methyltransferase DNMT1, blocking the Ras system from performing its important function of promoting cell apoptosis and inhibiting cell proliferation [131, 132] (Figs. 4 and 5, Table 1).

RGS7 and RGS11, also major members of the R7 family, can participate in the negative regulation of GPCRs and form a costable complex with the atypical G protein Gβ5. As a self-protection mechanism against myocardial fibrosis caused by the side effects of chemotherapy drugs, the expression of RGS7 and RGS11 in the heart increases after the patient receives chemotherapy, but the mechanism remains unelucidated [133, 134]. RGS7, initially localized as a tumour suppressor, is unstable in melanoma and prone to recurrent mutations, thus promoting the migration and invasion of melanoma cells, which may be related to the diminished activity of RGS7 in catalysing Gα-GTP hydrolysis and the instability of the protein itself [101]. Shenghui Yang et al. found that RGS11 is highly expressed in the lymph node and bone metastases of lung adenocarcinoma patients, but interestingly, the enhanced and diminished RGS11 expression revealed its specific role only in cell migration, and no correlation with cell invasion or proliferation has been reported [101]. Using the KM-plotter database, Yuexin Hu et al. found that RGS11 is overexpressed in ovarian cancer and promotes the development and progression of ovarian cancer, but the specific mechanism has not been explored [34] (Figs. 4 and 5, Table 1).

### RGS D/R12 subfamily

Unlike the other three subfamilies, the RGS D/R12 subfamily contains family members that vary widely, with RGS10 being a relatively simple RGS protein with a size of 20 kDa, while RGS12 and RGS14 are much larger and more complex than RGS10. RGS12 and RGS14 have a tandem RAS-binding domain (RBD) and a C-terminal GoLoco motif (GoLoco), which are guanine nucleotide dissociation inhibitors (GDIs) of the Gai/o-subunit [5] (Figs. 2 and 3D). Feyzanur Yildirimtepe et al. found higher expression of RGS10 in normal colorectal tissues than in tumour tissues and a negative correlation between DNA methylation and RGS10 transcripts [104]. In ovarian cancer cells, inhibition of RGS10 expression promotes the activation of the AKT signalling pathway, leading to enhanced cell proliferation, which in turn promotes the progression of ovarian cancer. Therefore, RGS10 may be one of the key targets for the treatment of cancer [105] (Fig. 4, Table 1).

RGS12 has additional N-terminal motifs, including the PSD-95/DLG/ZO1 (PDZ) structural domain and phosphotyrosine binding (PTB) structural domain, and is the classical RGS protein family member with the highest molecular weight (Fig. 3D). The PSD-95/DLG/ZO1 (PDZ) structural domain can bind to mitogen-activated protein kinase (MEK2), and the PTB structural domain (PTB) can bind to N-type calcium channels. RGS12 is involved in regulating a variety of important transmissions in the body, which is important for normal as well as tumour tissues and cells [128]. Yongquan Wang et al. found low RGS12 protein expression in prostate cancer tissues and cells obtained from African-Americans and demonstrated that RGS12, as a novel tumour suppressor, can inhibit the AKT and MNX1 signalling pathways [109]. RGS12 can also act as a tumour suppressor in osteosarcoma by inhibiting the expression and function of other relevant markers of osteosarcoma [111]. C Fu et al. demonstrated that knocking down RGS12 in oral squamous cell carcinoma significantly increased cancer cell proliferation and migration in transgenic mice and that RGS12 can inactivate the AKT/mTOR signalling pathway, thereby inhibiting tumour cell development [110] (Figs. 4 and 5, Table 1). Collectively, these findings indicate that RGS12 acts as a tumour suppressor and a novel promising target for the treatment of various cancers.

The biological impact of RGS8, RGS18 and RGS21 of the R4 subfamily, RGS9 of the R7 subfamily and RGS14 of the R12 subfamily on tumours and cancers has not been systematically reported. RGS13 of subfamily R4 has been used as a new specific marker only for condyloma lymphoma (MCL) in B lymphoma, and the mechanism of RGS13 in MCL has not been elucidated [85]. However, its potential impact on tumour/cancer cell generation and development and the high value of targeted cancer therapy will be further explored in the future.

## Conclusion and perspective

RGS proteins play a crucial role in cancer progression, and their roles in cancer are closely related to protein structure. All four RGS subgroups contain the RGS box (RH) signature motif and are involved in proliferation, apoptosis, migration, and kinase signalling in a variety of cancers, suggesting that this motif may contribute to their role in tumour kinase signalling. There are some structural differences between these four subgroups, and therefore, they also have some functional differences. Even members of the same subgroup show some functional differences; for example, RGS2 and RGS4 in the B/R4 subgroup have a low expression status in a variety of tumours [41, 49, 62, 63], whereas RGS1 and RGS5 have a high expression status in most tumour types [32, 35, 72, 73] (Table 1). This may be due to other structural differences that cause functional changes, which need to be further investigated.

Current studies of RGS proteins in cancer have focused on phenotypic factors, such as the inhibitory or promotional role of RGS in cell proliferation, apoptosis, necrosis, metastasis, and drug resistance [15, 17, 76, 81]. It is puzzling that although RGS proteins have similar conserved structural domains, some RGS proteins have pro-cancer properties, while others play opposite roles. For example, some RGS proteins, namely, RGS1, RGS3, RGS5, and RGS13, have tumour-promoting effects, whereas other proteins, namely, RGS2, RGS4, RGS6, RGS10, and RGS12 have tumour-suppressive effects. This may be due to undetected structural differences or specific structural changes in different tumours. In addition, depending on the tumour type, some RGS proteins play both tumour-suppressive and tumour-promoting roles, which may be the mechanism for their different roles in different tumour types. For example, RGS16 plays a tumour-promoting role and is a potential diagnostic marker in colorectal cancer [88], whereas it plays a tumour-suppressive role in breast cancer by inhibiting the PI3K signalling pathway [87]. RGS proteins may affect the activation or inactivation of a variety of kinases in mediating GPCR signalling. However, the kinases directly affected by RGS proteins identified to date remain unknown, and the specific molecular mechanisms of RGS proteins in cancer progression have not been comprehensively determined. Therefore, more in-depth studies are needed to clarify the exact functions of RGS proteins and explore their molecular mechanisms in cancer, to provide a theoretical basis for more effective cancer treatment. In addition, most RGS proteins have been reported to be associated with patient prognosis, suggesting that RGS proteins are potential biomarkers for cancer therapy.

It is well known that the development of efficient drugs for the treatment of cancer is essential. Currently, based on previous reports, the functional roles of RGS proteins in cancer also do not seem to have significant specificity among different tumour types. To further determine the specificity of the four subgroups of RGS in different human cancers, the researchers analysed the expression of RGS in a variety of cancers using online databases in conjunction with research reports. It was found that different RGS subpopulations have significant specificity for certain cancers (Table 1, Fig. 4), which may help in their diagnosis and treatment. In addition, due to the structural diversity of RGS proteins, it is very difficult to produce inhibitors of single RGS proteins. Therefore, further work is urgently needed to find new ways to produce efficient drugs targeting RGS proteins that can contribute to cancer therapy and influence drug development in other areas.

## Data Availability

Not applicable.
